# The efficacy of bandage contact lens in relieving the aggravation of dry eye disease after complicated cataract or/and IOL surgery

**DOI:** 10.1186/s12886-024-03385-x

**Published:** 2024-03-28

**Authors:** Dan Chen, Dejian Xu, Xingdi Wu, Jingwen Wang, Siting Sheng, Xuewen Yu, Xueqi Lin, Lirui Liu, Xian Ge, Huiling Zhao, Wen Xu

**Affiliations:** 1 Eye Center, The Second Affiliated Hospital, School of Medicine, Zhejiang Provincial Key Laboratory of Ophthalmology, Zhejiang Provincial Clinical Research Center for Eye Diseases, Zhejiang University, Zhejiang Provincial Engineering Institute on Eye Diseases, Hangzhou, Zhejiang China; 2 Lemolight Ophthalmology Hospital, Hangzhou, Zhejiang China; 3 Department of Ophthalmology, The First People’s Hospital of Xiaoshan District, Xiaoshan Affiliated Hospital of Wenzhou Medical University, Hangzhou, Zhejiang China; 4 Department of Ophthalmology, Zhongshan Hospital, Fudan University, Shanghai, China; 5 Jianyang Eye Hospital of Jianhu County, Jiangsu, China

**Keywords:** Complicated cataract or/and IOL surgery, Dry eye disease, Bandage contact lens, Retrospective

## Abstract

**Purpose:**

In the present study, we aimed to evaluate the efficacy of the bandage contact lens (BCLs) in the treatment of dry eye disease (DED) after complicated cataract or/and intraocular lens (IOL) surgery.

**Methods:**

In this retrospective, single-centered, observational study, we collected data from 69 patients who underwent complicated cataract or/and IOL surgery. Of these, 35 cases wore their own BCLs immediately after the operation, while the other 34 cases did not have their own BCLs and were instead covered with gauze. The Ocular Surface Disease Index (OSDI) questionnaire, slit-lamp microscope examination, keratograph analysis, and Schirmer I test were measured at baseline, 1 week and 1 month postoperatively.

**Results:**

In the BCL group, the score of the OSDI questionnaire was significantly decreased at 1 week and 1 month postoperatively compared with baseline levels (*P* = 0.000, collectively). Moreover, the fluorescein staining score of the BCL group was remarkably decreased 1-week and 1-month postoperatively compared with the non-BCL group (*P* = 0.000 and *P* = 0.000, respectively). Furthermore, the redness score of the BCL group was also better compared with the non-BCL group at 1 week and 1 month postoperatively (*P* = 0.014 and *P* = 0.004, respectively).

**Conclusions:**

Complicated cataract or/and IOL surgery would intensify the DED. Early application of BCLs postoperatively improved patients’ comfort and alleviated dry eye-related symptoms and signs. Furthermore, this mechanism might involve the acceleration of corneal epithelial healing, the alleviation of ocular stress response and the stabilization of the tear film.

**Trial registration:**

Trial registration ClinicalTrials, NCT04120389. Registered 10 October 2019—retrospectively registered.

## Background

Phacoemulsification remains the most widely used and effective ophthalmic surgery to improve the visual quality of patients [1]. Although a better visual acuity can be obtained, most patients are still unsatisfied with the visual experience accompanied by ocular discomfort [2]. Several studies have demonstrated the association between cataract surgery and the development or exacerbation of dry eye disease (DED) [3]. As a multifactorial disease, DED can induce ocular discomfort due to the abnormality of the tear film and ocular surface [4, 5]. Compared with simple phacoemulsification, complicated cataract or/and intraocular lens (IOL) surgeries are always integrated with more anterior eye segment operations, such as anterior vitrectomy, capsular tension ring implantation, IOL scleral fixation, and iris repair. Patients accepting complicated cataract or/and IOL surgeries face longer surgery duration accompanied by more prolonged exposure to intraoperative illumination, more anesthetics, greater surgical injury, irritation of the sutures and longer use of topical medication postoperatively. As a result, the proportion of patients complaining of these ocular symptoms after complicated cataract or/and IOL surgery is also higher than simple cataract surgery.

Bandage contact lens (BCLs) have been widely applied to some corneal diseases and postoperative treatment after ocular-surface operations [6–8]. Previous research has brought BCLs in cataract surgery to alleviate postoperative dry eye-related symptoms [9–11]. However, only very few studies have focused on the status and treatment of DED after complicated cataract or/and IOL surgery.

In this study, we aimed to assess the effectiveness of BCLs in alleviating dry eye-related symptoms and signs following complicated cataract and/or IOL surgery.

## Patients and methods

This retrospective study was approved by the Ethics Committee of the Second Affiliated Hospital, School of Medicine, Zhejiang University, and registered at http://www.clinicaltrials.gov (identification no. NCT04120389). Moreover, this study complied with the tenets of the World Medical Association of Helsinki.

Diagnosis of DED met TFOS DEWS II diagnostic criteria, which required subjective symptoms [Ocular surface disease index (OSDI) ≥ 13] and at least one of the following results: (i) fluorescein break-up time (F-BUT) < 10 s, and (ii) the fluorescein staining score > 5 corneal spots [12].

Dry eye severity can be classified into three categories: (i) Mild: fluorescein staining spots less than 5 and F-BUT of 2 s or more. Defined as level 0. (ii) Moderate: fluorescein staining spots of 6 to 30, with the F-BUT of 2 s or more. Defined as level 1. (iii) Severe: fluorescence staining spots of 30 or more, with a BUT of less than 2 s. Categorized as level 2.

### Study population and procedures

#### Inclusion criteria

DED was diagnosed preoperatively, and complicated cataract or/and IOL surgery was required.

Complicated cataract or/and IOL surgery involves the use of multiple surgical techniques performed simultaneously, which may include: (1) phacoemulsification or/and IOL implantation, (2) anterior vitrectomy, (3) IOL scleral fixation, (4) capsular tension ring implantation with/without suture fixation and (5) iris repair or pupilloplasty.

#### Exclusion criteria

1. Patients with immune system diseases (systemic immunosuppressants or long-term hormone use) and diabetes.
2. Patients who postoperative lost-to-follow-up.

### Ophthalmological assessments

OSDI questionnaire was adopted to assess subjective dry eye-related feelings. The OSDI questionnaire consisted of 12 questions and was divided into three sections as follows: (1) ocular subjective symptom subscore (items 1 to 5), (2) visual function subscore (items 6 to 9), and (3) environment-related visual subscore (items 10 to 12). Finally, the total score was calculated using the formula: (total score of all answered questions x100) / (total number of answered questions x 4).

Fluorescein breakup time (F-BUT) was performed to assess tear film stability. Briefly, a single fluorescein strip (Meizi, Liaoning, China) was placed in the conjunctival sac of the eye after instilling a drop of normal saline, and the patient was asked to stare straight ahead without blinking. The time from the last blink to the first appearance of a randomly distributed dry spot was measured in triplicate, and the mean value was recorded.

Corneal fluorescein staining was performed as previously described. If there were no punctate epithelial erosions (PEEs), the score was 0. If one to five PEEs were seen, the corneal score was 1, six to thirty PEEs were scored as 2, and > thirty PEEs were scored as 3. An additional point was added if (1) PEE occurred in the central 4-mm diameter portion of the cornea, (2) one or more filaments, or (3) one or more patches of confluent staining, including linear stains. The maximum possible score for each cornea was 6 [13].

The keratography analysis was performed by using a keratograph (Oculus, Wetzlar, Germany) to determine the bulbar redness score, tear meniscus height (TMH), noninvasive first break-up time (NIF-BUT), noninvasive average break-up time (NIAvg-BUT) and meibography structure. 

The redness score was determined based on the area percentage ratio of blood vessels in the bulbar conjunctiva, which indicated the inflammatory condition.

TMH below the central pupil was determined by the keratography analysis to assess the secretion of tears and the patency of the lacrimal ductal system.

The degree of meibography losses was scored on the following scale: 0 (no loss of meibomian glands); 1 (area loss less than one-third of the total meibomian gland area); 2 (area loss between one-third and two-thirds of the total meibomian gland area); and 3 (area loss more than two-thirds of the total meibomian gland area). The total score was the sum of the scores of the upper and lower eyelids and was recorded as 0 to 6.

Schirmer I test was performed to assess the basic tear secretion function. The Schirmer tear test strip (Meizi, Liaoning, China) was placed in the middle of the lower eyelid, and the length of the test strip was recorded 5 min later.

### Data collection

The study observation period spanned from the date the patient started receiving care at the study site until the last data point was recorded. All these ocular data were collected before the surgery and at 1 week and 1 month postoperatively.

### Complicated cataract or/and IOL surgery

Preoperatively, levofloxacin 0.5% (Santen, Osaka, Japan) was instilled four times daily for 3 days. Pupillary dilation was achieved with one drop of tropicamide every 15 min, three times before surgery. The anesthesia method was parabulbar injection. All cataract or/and IOL surgeries were performed by the same ophthalmologist (X.W.).

The BCLs (Air OptixNight &Day Aqua-Soft Contact Lenses) were worn by the surgeon on the operating table to ensure sterility at the end of surgeries and removed by doctors at a 1-week follow-up postoperatively. Patients who did not have their own BCLs were covered with sterile gauze on the operating table by doctors.

Postoperatively, all patients were instructed to take methylprednisolone tablets (Pfizer Italia Srl, AP, Italy) orally at a dose of 12 mg per day for 3 days. Additionally, prednisolone acetate ophthalmic suspension (Allergan, Co., Mayo, Ireland), levofloxacin 0.5% (Santen, Osaka, Japan), and diclofenac sodium 1% (Qiyan, Shenyang, China) were instilled four times daily for 1 week after surgery. In the BCL group, while wearing the BCL, the eye drops were dropped into the fully exposed lower conjunctival sac after gently pulling the lower eyelid downwards. At the 1-week follow-up, the medication prescription was changed to 0.1% pranoprofen (Senju Pharmaceuticals, Kobe, Japan) and carbomer eye gel (Dr. Gerhard Mann, Berlin, Germany) four times daily until consumption.

The corresponding number of surgical techniques among the 69 patients included in the study is shown in Table 1.

**Table 1:** Surgical techniques involved in this study

	Non-BCL group	BCL group
phacoemulsification or/and IOL implantation	20	16
anterior vitrectomy	31	32
IOL scleral fixation	21	15
capsular tension ring implantation or/and suture fixation	3	9
iris repair or pupilloplasty	3	11

### Statistical analysis

Statistical analysis was performed using SPSS software (version 22.0, SPSS, Inc., Chicago, IL). The Chi-square test was used to compare the sex and laterality between the two groups. Normal distribution was confirmed using the independent-sample t-test to compare the differences between the two groups. Otherwise, the Mann-Whitney Utest was used. A repeated measures analysis of variance (ANOVA) test was performed to compare changes at all time points. Correlations between parameters were tested with the Pearson correlation coefficient (r). A *P* value of less than 0.05 was considered statistically significant.

## Results

**Fig. 1:**
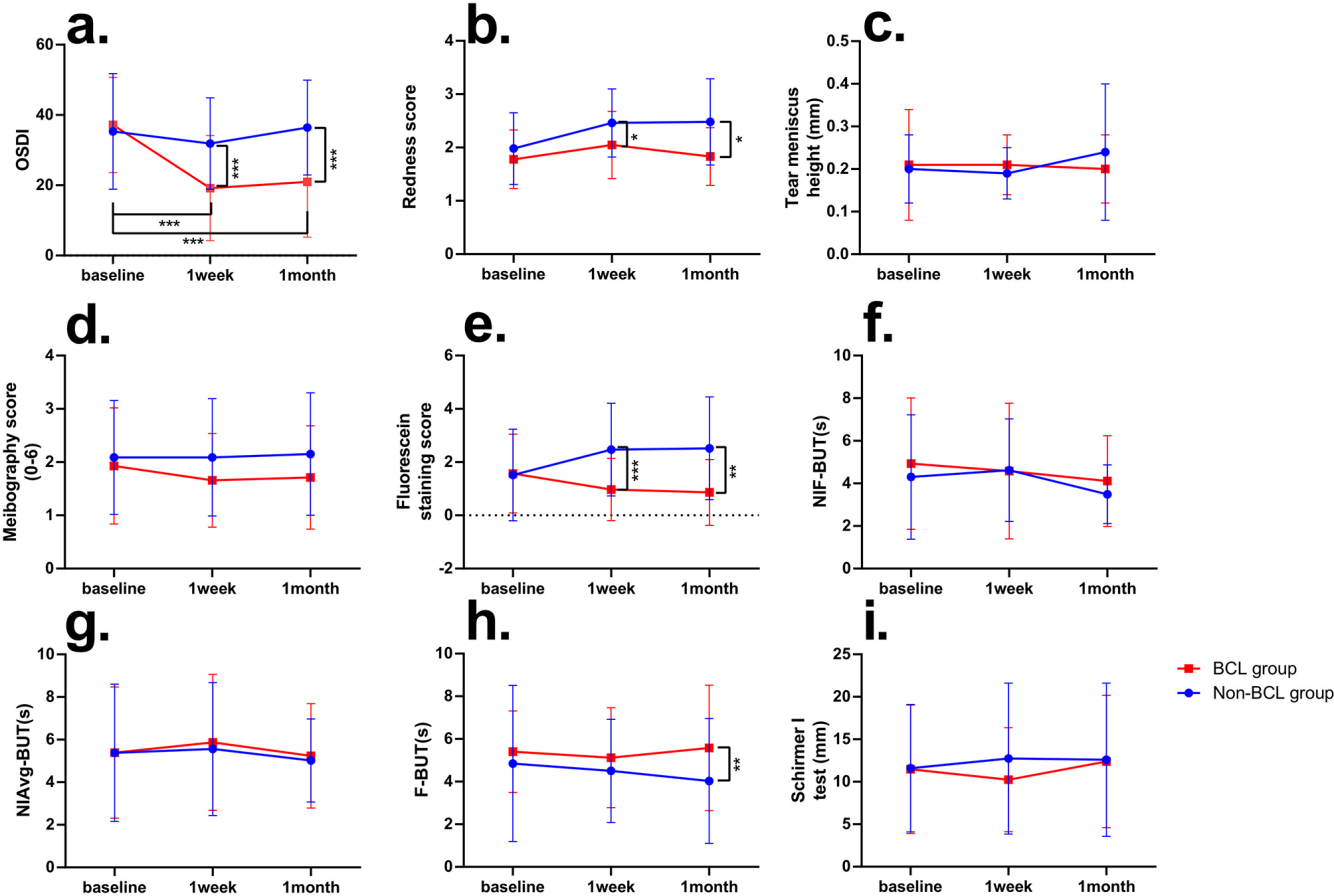
Changes in clinical signs and symptoms between the two groups over time. (**a**) Ocular Surface Disease Index (OSDI); (**b**) Bulbar redness score; (**c**) Tear meniscus height (TMH); (**d**) Meibography score; (**e**) Fluorescein staining scores; (**f**) Noninvasive first breakup time (NIF-BUT); (**g**) Noninvasive average breakup time (NIAvg-BUT); (**h**) Fluorescein breakup time (F-BUT); (**i**) Schirmer I test. *P<0.05, **P<0 0.01,***P<0.001

A total of 69 patients (69 eyes) were enrolled in this study. Among the participants in the BCL group, no adverse events (such as infection or inflammatory keratitis, conjunctivitis, corneal abrasions, neovascularization or any other events) were found and recorded. Table 2 shows the demographics and clinical features. No statistical differences were observed between the two groups regarding demographics and ocular surface parameters. Table 3 shows the comparison of preoperative and postoperative clinical dry eye-related parameters of both groups. A significant difference was noticed in the OSDI score between the two groups from the 1-week follow-up to the 1-month follow-up (Fig. 1a). In the BCL group, the OSDI score postoperatively was statistically lower compared with baseline (*P* < 0.001, collectively) (Table 3).

**Table 2:** Demographics and clinical features in this study

Parament	Non-BCL group	BCL group	*P* Value
Number of eyes	34	35	
OD(%)	14 (41.17%)	22 (62.85%)	0.118
Female (%)	5 (14.70%)	8 (22.85%)	0.381
Age (range)	57.41 ± 11.86 (18–85)	56.34 ± 14.49 (23–80)	0.739
OSDI (0-100)	35.34 ± 16.46 (*n* = 33)	37.16 ± 13.54 (*n* = 35)	0.469
Redness score (1–4)	1.98 ± 0.67 (*n* = 29)	1.78 ± 0.55 (*n* = 28)	0.221
TMH (mm)	0.20 ± 0.08 (*n* = 33)	0.21 ± 0.13 (*n* = 34)	0.518
Meibography score	2.09 ± 1.07 (*n* = 33)	1.93 ± 1.09 (*n* = 29)	0.731
FL	1.52 ± 1.72 (*n* = 34)	1.57 ± 1.48 (*n* = 35)	0.782
NIF-BUT (s)	4.30 ± 2.92 (*n* = 31)	4.93 ± 3.08 (*n* = 35)	0.403
NIAvg-BUT (s)	5.38 ± 3.22 (*n* = 31)	5.39 ± 3.08 (*n* = 35)	0.994
F-BUT (s)	5.08 ± 3.86 (*n* = 34)	5.40 ± 1.91 (*n* = 34)	0.103
Schirmer I test	11.60 ± 7.50	11.47 ± 7.56	0.847
Dry eye severity grade (0–2)	0.76 ± 0.92	0.80 ± 0.83	0.758

OSDI Ocular Surface Disease Index; TMH tear meniscus height; FL Fluorescein staining score; NIF-BUT noninvasive first breakup time; NIAvg-BUT noninvasive average breakup time; F-BUT Fluorescein breakup time. Data are presented as means ± standard deviation (SD)

**Table 3:** Statistical comparison of preoperative and postoperative dry eye-related parameters of both groups

Parameter	Time	Non-BCL group (*n* = 34)		BCL group (*n* = 35)		*P* Value
		Mean ± SD	Range	Mean ± SD	Range	
OSDI (0—100)	Baseline	35.34 ± 16.46 (*n* = 33)	13.63, 75.00	37.16 ± 13.54 (*n* = 35)	14.58, 70.00	0.469
	1 week	31.87 ± 13.02 (*n* = 33)	2.77, 62.50	19.17 ± 14.96 (*n* = 35)	0.00, 66.66	0.000***
	1 month	36.42 ± 13.49 (*n* = 25)	10.00, 65.00	20.97 ± 15.75 (*n* = 29)	0.00, 59.09	0.000***
Redness score	Baseline	1.98 ± 0.67 (*n* = 29)	0.90, 3.30	1.78 ± 0.55 (*n* = 28)	0.70, 3.20	0.221
	1 week	2.46 ± 0. 64 (*n* = 29)	1.30, 3.80	2.05 ± 0.63 (*n* = 32)	1.20, 3.50	0.014*
	1 month	2.48 ± 0.81 (*n* = 23)	1.10, 4.00	1.83 ± 0.54 (*n* = 28)	1.00, 2.80	0.004*
TMH (mm)	Baseline	0.20 ± 0.08 (*n* = 33)	0.09, 0.53	0.21 ± 0.13 (*n* = 34)	0.11, 0.90	0.518
	1 week	0.19 ± 0.06 (*n* = 33)	0.05, 0.38	0.21 ± 0.07 (*n* = 35)	0.10, 0.38	0.551
	1 month	0.24 ± 0.16 (*n* = 26)	0.10, 0.97	0.20 ± 0.08 (*n* = 29)	0.07, 0.51	0.483
Meibography score (0–6)	Baseline	2.09 ± 1.07 (*n* = 33)	0.00, 4.00	1.93 ± 1.09 (*n* = 29)	0.00, 4.00	0.731
	1 week	2.09 ± 1.10 (*n* = 33)	0.00, 4.00	1.66 ± 0.88 (*n* = 33)	0.00, 3.00	0.166
	1 month	2.15 ± 1.15 (*n* = 26)	0.00, 4.00	1.71 ± 0.97 (*n* = 28)	0.00, 3.00	0.221
FL	Baseline	1.52 ± 1.72	0.00, 5.00	1.57 ± 1.48	0.00, 5.00	0.782
	1 week	2.47 ± 1.74 (*n* = 34)	0.00, 6.00	0.97 ± 1.17	0.00, 3.00	0.000***
	1 month	2.52 ± 1.93 (*n* = 25)	0.00, 5.00	0.86 ± 1.24 (*n* = 29)	0.00, 4.00	0.001**
NIF-BUT(s)	Baseline	4.30 ± 2.92 (*n* = 31)	1.15, 15.29	4.93 ± 3.08 (*n* = 35)	1.02, 14.34	0.403
	1 week	4.62 ± 2.41 (*n* = 31)	1.02, 11.28	4.58 ± 3.18 (*n* = 35)	1.21, 13.96	0.266
	1 month	3.49 ± 1.38 (*n* = 24)	1.27, 6.88	4.11 ± 2.13 (*n* = 29)	2.48, 13.45	0.171
NIAvgBUT (s)	Baseline	5.38 ± 3.22 (*n* = 32)	1.15, 15.29	5.39 ± 3.08 (*n* = 35)	1.02, 14.34	0.994
	1 week	5.56 ± 3.12 (*n* = 32)	0.10, 13.93	5.87 ± 3.19 (*n* = 35)	1.21, 14.10	0.688
	1 month	5.02 ± 1.95 (*n* = 24)	1.27, 9.47	5.24 ± 2.45 (*n* = 29)	2.74, 13.98	0.872
F-BUT (s)	Baseline	4.85 ± 3.66 (*n* = 34)	0.00, 15.00	5.40 ± 1.91 (*n* = 34)	2.00, 9.00	0.103
	1 week	4.50 ± 2.42 (*n* = 32)	1.00, 12.00	5.12 ± 2.34 (*n* = 35)	1.00, 13.00	0.249
	1 month	4.03 ± 2.93 (*n* = 25)	0.00, 16.00	5.58 ± 2.94 (*n* = 29)	1.00, 14.00	0.009**
Schirmer I test (mm)	Baseline	11.60 ± 7.50	2.00, 30.00	11.47 ± 7.56	2.00, 30.00	0.847
	1 week	12.73 ± 8.88	2.00, 35.00	10.24 ± 6.13	1.00, 24.00	0.387
	1 month	12.60 ± 9.02 (*n* = 25)	2.00, 30.00	12.39 ± 7.79 (*n* = 29)	2.00, 35.00	0.761
Dry eye severity grade (0–2)	Baseline	0.76 ± 0.92 (0.00,2.00)		0.80 ± 0.83 (0.00,2.00)		0.758
	1 week	1.21 ± 0.84 (0.00,2.00)		0.51 ± 0.74 (0.00,1.00)		0.001**
	1 month	1.24 ± 0.93 (0.00,2.00) (*n* = 25)		0.45 ± 0.74 (0.00,1.00) (*n* = 25)		0.002**

OSDI Ocular Surface Disease Index; TMH tear meniscus height; FL Fluorescein staining score; NIF-BUT noninvasive first breakup time; NIAvg-BUT noninvasive average breakup time; F-BUT Fluorescein breakup time. Data are presented as means ± standard deviation (SD). *P < 0.05, **P < 00.01, ***P < 0.001

A statistically significant difference was detected in the redness score of both groups at 1-week and 1-month follow-up (*P* = 0.014 and *P* = 0.004, respectively; Fig. 1b). Moreover, the redness score in the BCL group returned to the baseline level at the 1-month follow-up, while that of the non-BCL group remained higher, leading to a significant discrepancy between the two groups (Table 4).

**Table 4:** Statistical analysis of preoperative and postoperative clinical signs in both groups

	*P* Value					
	Non-BCL group (34)			BCL group (35)		
	B vs1W	B vs. 1 M	1 M vs. 1 W	B vs. 1 W	B vs. 1 M	1 M vs. 1 W
OSDI	0.580	0.861	> 0.99	0.000**	0.000***	> 0.99
Redness score	0.130	0.254	> 0.99	0.428	> 0.99	0.329
TMH (mm)	0.960	> 0.99	0.826	> 0.99	0.913	0.487
Fluorescein staining score	0.059	0.344	> 0.99	0.114	0.335	> 0.99
NIF-BUT (s)	0.323	> 0.99	0.052	> 0.99	> 0.99	> 0.99
NIAvg-BUT (s)	> 0.99	> 0.99	0.807	> 0.99	> 0.99	> 0.99
F-BUT (s)	> 0.99	> 0.99	> 0.99	> 0.99	> 0.99	> 0.99
Schirmer I test (mm)	> 0.99	> 0.99	> 0.99	> 0.99	> 0.99	> 0.99

OSDI Ocular Surface Disease Index; TMH tear meniscus height; FL Fluorescein staining score; NIF-BUT noninvasive first breakup time; NIAvg-BUT noninvasive average breakup time; F-BUT Fluorescein breakup time. Data are presented as means ± standard deviation (SD). *P < 0.05, **P < 00.01, ***P < 0.001

The change in fluorescein staining score of the non-BCL group was significantly more than the BCL group at 1-week and 1-month follow-up (*P* = 0.000 and *P* = 0.001, respectively; Fig. 1e).

At 1-month follow-up, the F-BUT of the BCL group was statistically longer compared with the non-BCL group (*P* = 0.007; Fig. 1h).

The dry eye severity of the two groups after surgery had significant statistical differences, and the BCL group was lighter than the non-BCL group (Table 3).

There was no statistical difference in NIAvg-BUT, Schirmer I test, TMH and meibography gland structure evaluation during the follow-up time (Fig. 1c,d,f,g, i).

## Discussion

BCLs have been applied in a wide variety of ocular surface disorders, such as Fuchs’ dystrophy, toxic epitheliopathy, filamentous keratitis, corneal perforation, corneal stromal melting, recurrent corneal erosion, chemical or traumatic corneal epithelial defects, epithelial irregularity, persistent epithelial defects, lamellar laceration, graft insufficiency and pseudophakic bullous keratopathy [14–16]. In addition, BCLs can be used immediately after ocular refractive surgeries, such as PRK [17], LASIK and corneal crosslinking [18], to aid in epithelial healing and relieve postoperative pain and discomfort. The BCLs (Air Optix Night & Day Aqua-Soft Contact Lenses) are made of silicone hydrogel. Compared with conventional hydrogels, silicone hydrogels possess higher oxygen transmission and higher modulus values, which help corneal wounds recover and increase wearing comfort. Additionally, silicone hydrogel contact lenses may help stabilize the tear film, allow for corneal healing and restore normal cell turnover, all of which are critical in the treatment of DED [14].

Development of DED after cataract surgery is multifactorial, including corneal nerve transection, prolonged microscopic light exposure, elevation of inflammatory factors, goblet cell loss, and meibomian gland dysfunction [19]. Compared with simple micro-incision phacoemulsification, complicated cataract or/and IOL surgery requires suture procedures involving more corneal incisions. Larger and more incisions take a longer time to recover [20]. In addition, longer operating time means prolonged microscopic light exposure. Additional corneal conjunctiva sutures can also cause discomfort and abnormal sensation [21]. Surgical incisions will disrupt the normal organization of corneal innervation. Denervation of the cornea results in impaired epithelial wound healing, increased epithelial permeability, decreased epithelial metabolic activity, and loss of cytoskeletal structures associated with cellular adhesion [20, 22]. Eventually, the tear flow and blink rate are reduced, leading to the instability and hyperosmolarity of the tear film [23]. Additionally, complicated cataract or/and IOL surgery takes a longer time and causes larger tissue injury which promotes a more serious inflammatory reaction [9]. Some researchers have implicated that inflammation is closely related to the aggravation of DED [24]. Dan-Na Shi et al [11]. have reported the safety of therapeutic BCLs on post-cataract surgery patients. Previous studies have proved that proper use of BCLs effectively alleviates DED after cataract surgery [9, 10]. Based on the excellent properties of BCLs, we aimed to confirm the efficacy of BCLs in easing deterioration of DED after complicated cataract or/and IOL surgeries.

Our present study observed that dry eye-related signs worsened postoperatively in the non-BCL group and did not recover to the baseline at the 1-month follow-up. This finding was consistent with previous research [2]. The dry eye severity grade of the non-BCL group showed a deterioration trend. Compared with the non-BCL group, several corresponding dry eye-related parameters in the BCL group were significantly improved at the same time point.

The OSDI score is a valid and reliable instrument for measuring the severity of DED [25]. We noticed that the OSDI score of the BCL group was significantly lowered at 1-week and 1-month reviews from the baseline (*P* < 0.000, collectively). Besides, the postoperative OSDI score was also statistically different between the two groups, suggesting that the application of BCLs greatly improved subjective dry-eye symptoms and enhanced patients’ comfort. Kyoung Yul Seo et al. [26] have found that the symptoms of DED after the cataract surgery are related to the depressive and anxiety states of patients. In addition to the air permeability and excellent performance of BCLs, the existence of BCLs also brings comfort of mind to patients to a certain extent, in terms of resisting external infection and promoting wound healing.

Previous studies have proved that BCLs can accelerate the process of corneal re-epithelialization [27]. Based on this function, BCLs are widely applied after pterygium and PRK surgery [18]. Ding Chen et al. have used UHR-OCT to prove the efficacy of BCLs on corneal epithelial healing [28]. However, fluorescein staining is still the most simple and direct method to evaluate corneal defects in clinical practice. In our present study, the fluorescein scores of the BCL group at 1 week and 1 month after surgery were meaningfully lower compared with the baseline levels (*P* = 0.001 and *P* = 0.002, respectively). The other thing we could observe was that the corneal condition of the non-BCL group was inferior to the BCL group at the same time point. The BCLs cover the corneal surface, providing a well-constructed repair space for corneal epithelial injury, and promoting the regeneration of corneal epithelial cells [7]. Moreover, its regular intra-limbal surface will provide the cornea with a protective layer of tear fluid and high oxygen levels through the tear-pump mechanism and the use of a high Dk material [29].

It was observed that there was no difference in the meibography score in both groups compared with the baseline in our study. This finding was consistent with previous studies that meibomian gland function may be altered without accompanying structural changes after cataract surgery [30].

Bulbar redness is a non-specific ocular response due to vasodilation of the conjunctival and/or anterior scleral blood vessels, indicating enhanced blood flow to the anterior ocular tissues [31]. Conditions known to induce bulbar redness include anterior eye inflammation, allergic and infective conjunctivitis, contact lens wear, meibomian gland dysfunction, and DED [32]. Earlier studies have supposed that the redness score is linked to the wear of contact lenses. The redness level is related to the extent of oxygen transmissibility of contact lens materials [33, 34]. Laura E. Downie [32]has confirmed that the accuracy of analyzing bulbar redness using an ocular surface comprehensive analyzer surpassed the evaluations conducted by clinicians with relevant scales. In our present study, the redness scores of the non-BCL group were significantly increased at 1 week after surgery from the baseline level and remained higher at 1-month follow-up, indicating that complicated cataract or/and IOL surgery might intensify bulbar redness. The reason might be that a series of invasive operations during the prolonged surgery damaged body tissue and induced surgical stress, leading to the release of a substantial amount of inflammatory factors and consequent ocular surface inflammation [35]. Corneal sutures also irritated it. Furthermore, there was a significant difference between the two groups postoperatively. The swift recovery of the redness scores in the BCL group was shown. The BCLs could inhibit the precipitation of corneal metabolites, improving the safety of wearing them. The covering effect of BCLs could alleviate the friction between the corneal suture, conjunctival suture and conjunctiva, as well as the mutual physical stimulation between nerve endings and eyelids [7].

Due to complicated cataract or/and IOL surgery involving multiple corneal incisions, there is a risk of infection in the surgical wound. Therefore, the BCLs must be applied by the primary surgeon in a sterile operating condition. The prescription and wearing of the BCLs should be strictly in accordance with the doctor’s instructions.

Schirmer I test reflects the basic tear secretion and tear-film volume, which specifically evaluates the aqueous layer of the tear film and TMH [36]. The result of the Schirmer test in the non-BCL group showed a transient increase at 1 week, while that in the BCL group was decreased. The possible alternative explanation for this observation was that surgical-induced and suture-correlated pain and irritation to the ocular surface might contribute to the artificially higher tear lake. The existence of BCLs promoted corneal wound healing, which reduced the exposure of corneal nerve endings and thus reduced pain. In the follow-up of 1 month, the results of the Schirmer test of both groups tended to the preoperative level. There was no significant difference in tear volume between the two groups. The noninvasive measurement of TMH conducted by the keratograph was not correlated well with the Schirmer test score (*r* = 0.143, *P* = 0.096 by the Spearman correlation test). This finding was inconsistent with Yinhui Yu et al.’s study [37]. The possible explanation was that complicated cataracts involve multiple corneal incisions, and the injured nerve endings are not sensitive to the stimulation of Schirmer I test paper.

As for the results of NIAvg-BUT, a brief rise could be observed at 1 week postoperatively in both groups, while it fell at 1-month follow-up, reflecting the unstable tear film postoperatively. The decrease of the BUT suggested the destabilization of the tear film [38]. Surprisingly, the F-BUT of the BCL group was significantly better compared with the non-BCL group at 1 month postoperatively. Although there might exist a certain subjective error, it also showed the role of BCLs in improving the stability of tear film to a certain extent.

Our study had some limitations. The main limitation of the present study is the small sample size. Secondly, due to the wide variety of complicated cataract or/and IOL surgery, it was difficult to completely match the surgical operations of the BCL group and the non-BCL group. Finally, for those undergoing complicated cataract or/and IOL surgery, it might require a longer recovery time.

In conclusion, our study found that complicated cataract or/and IOL surgery would intensify the DED. Early application of BCLs improved patient comfort and alleviated dry eye-related symptoms and signs. This possible mechanism might involve the acceleration of corneal epithelial healing, the alleviation of ocular stress response and the stabilization of the tear film.

## Data Availability

The datasets generated and analyzed during the current study are not publicly available due to Subsequent series of studies but are available from the corresponding author on reasonable request.
